# The relationship between overweight or obesity, elevated blood pressure and the overall quality of life in medical students, Poland

**DOI:** 10.3389/fpubh.2026.1922706

**Published:** 2026-09-17

**Authors:** Iwona Zieleń-Zynek, Szymon Szemik, Małgorzata Kowalska

**Affiliations:** Department of Epidemiology, School of Medicine in Katowice, Medical University of Silesia in Katowice, Katowice, Poland

**Keywords:** blood pressure, longitudinal study, medical students, obesity, quality of life, WHOQOL-BREF

## Abstract

**Introduction:**

Medical students are exposed to chronic stress, unhealthy lifestyle habits, and emerging cardiometabolic risks, which are usually related to their nutritional status and quality of life (QoL). This study aimed to assess the relationship between overweight or obesity, elevated blood pressure, and QoL among medical students in Poland, and to identify its determinants over a 2-year longitudinal period.

**Materials and methods:**

A total of 887 first-year medical students from the Medical University of Silesia in Katowice were enrolled in the prospective POLLEK study, with 705 students followed up in their second year. Anthropometric measurements, blood pressure assessments, and QoL evaluation using the WHOQOL-BREF questionnaire were performed at both time points. Logistic regression and chi-square tests were applied to identify predictors of lower QoL and elevated blood pressure across BMI categories.

**Results:**

The prevalence of overweight or obesity increased slightly from 12.9% in the first year to 13.6% in the second year, and was statistically significant higher among male students. A small but statistically significant increase in BMI and blood pressure values was observed over the follow-up period. The highest rates of elevated blood pressure were found among obese students at both time points (*p* < 0.0001). Overall QoL scores did not differ significantly across BMI categories. Logistic regression identified poor financial situation (OR = 1.669–1.910) and a history of chronic disease (OR = 2.153–2.228) as independent predictors of lower QoL in both years of observation.

**Conclusion:**

Overweight or obesity among medical students slightly increased over 2 years of follow-up and was associated with elevated blood pressure and unfavorable lifestyle patterns. The primary determinants of reduced QoL were financial hardship and chronic diseases rather than BMI itself. We believe that the early preventive interventions addressing weight disorders and health-promoting behaviors should be useful in the improvement of the situation.

## Introduction

To enhance the resilience of the healthcare system in Poland, a planning process for the development of medical staff has been initiated. One notable outcome of this effort is that the number of medical students has doubled between 2012 and 2023 (1). Medical studies are inherently dynamic and stressful, what can lead to negative changes in both living conditions and daily habits. Factors such as changing living environments, the need for greater accountability, and the adaptation to new educational methods and academic curricula can contribute to the occurrence of chronic stress among students (2).

The negative effects of stress on medical students can result in serious health issues. These consequences stem from factors such as a daily diet high in fast food, insufficient physical activity, substance abuse, including alcohol and tobacco use (3–5). The consequences of consistently poor eating habits and an unhealthy lifestyle can be seen in nutritional status issues, particularly when evaluated using Body Mass Index (BMI). A BMI value of 25 kg/m^2^ or higher is classified as overweight, while obesity is defined as a BMI of 30 kg/m^2^ or more in adults (6). According to the latest definition by Rubino et al., obesity can be categorized into two types: preclinical and clinical. Preclinical obesity is characterized by an excessive accumulation of adipose tissue and an increased risk of non-communicable diseases, such as type 2 diabetes, hypertension, certain types of cancer, and mental disorders. In contrast, clinical obesity is defined as a chronic, systemic disease. It manifests as dysfunction in tissues, organs, and potentially the entire body, resulting from excessive accumulation of adipose tissue (7, 8). Research studies have reported a positive correlation between obesity and elevated blood pressure in both men and women. It’s important to note that hypertension can be diagnosed in adults, the older adults, children, and adolescents, especially as obesity rates continue to rise (9). Young adults, including medical students, are at risk of experiencing abnormal blood pressure, which is a significant predictor of long-term cardiovascular disease. In response to this concern, the European Society of Cardiology (ESC) has updated its guidelines to include a new category: elevated blood pressure. This category is defined as having a systolic blood pressure between 120 mmHg and 139 mmHg, or a diastolic blood pressure between 70 mmHg and 89 mmHg (10, 11). Considering the various factors linked to medical studies – such as chronic stress, a diet low in fiber (like fruits and vegetables), high in saturated fatty acids, low physical activity, a tendency toward substance use, and low social status–we can evaluate how these elements contribute to visceral obesity, declared hypertension, and other chronic diseases. This understanding provides a foundation for assessing the quality of life of medical students.

Quality of life (QoL) is a multidimensional concept that includes physical health, psychological well-being, social relationships, and environmental factors. In an academic setting, health-related quality of life (HRQoL) is particularly important as it reflects individuals’ perceptions of their physical and mental functioning (12, 13). Medical students often face prolonged exposure to stressful events, unhealthy lifestyle choices, and emerging cardiometabolic risks, which can negatively impact both physical and mental aspects of QoL. Issues such as reduced physical functioning, fatigue, sleep disturbances, symptoms of anxiety and depression, and impaired social interactions can lead to decreased life satisfaction and overall well-being. Therefore, monitoring QoL of medical students, particularly regarding overweight, obesity, and elevated blood pressure, can help to identify high-risk groups and guide the development of targeted preventive interventions (14).

The World Health Organization (WHO) emphasizes the importance of diet quality indicators–specifically nutrient adequacy, macronutrient balance, dietary diversity, and moderation in food consumption–in relation to cardiovascular risk factors. They have provided current recommendations for a healthy diet, which include guidelines on the quantity, quality, and variety of food consumed, as well as the necessity of engaging in regular physical activity (150 min each week) (2, 15). Following these recommendations can help to maintain and improve health, leading to a positive impact on overall QoL.

There is ongoing concern that medical students may continue to engage in unhealthy behaviors, which could increase their risk of being overweight or obese, leading to chronic diseases. Therefore, this study aimed to assess the relationship between overweight or obesity, hypertension and quality of life among medical students in Poland, as well as the factors that influence this relationship over time.

It is important to add that this is a continuation of the results presentation from the POLLEK study. We would like to underscore that our analysis was based on the new ESC classification (11). In addition, we investigated the association between the nutritional status of medical students and these new ESC blood pressure definitions. Furthermore, our goal was to verify previously published data by Szemik et al. (16) across the completed study group. Based on our findings, we were able to verify the consistency of the results.

## Materials and methods

Polish medical students (Medical University of Silesia in Katowice) were examined twice, in their first and second academic years, as part of the follow-up POLLEK (POLski LEKarz) study. The study group comprised first-year university students from the 2021/2022 and 2022/2023 cohorts. Data on lifestyle, socioeconomic situation, and quality of life were collected using an integrated questionnaire. Additionally, each student was measured and weighed, and blood pressure was measured in a standard manner. Detailed methods were described in our previous publication (16). The bioethics commission approved the study (No. KNW/0022/KB/217/19), and the project was granted by the Medical University of Silesia (No. BNW-1-042/K/4/O).

Quality of life was assessed using the Polish paper version of the WHOQOL-BREF questionnaire, whose scores ranged from 0 to 100. Determinants of QoL related to lifestyle, health status, or socioeconomic status were gathered through a self-reported questionnaire. A better overall QoL was observed for scores equal to or greater than the median value in the studied group (Me = 75). A Body Mass Index (BMI) of equal or over 30 was used to identify obesity, while a BMI of 25 or higher was used to indicate overweight, value of BMI 18.5 to 24.9 was interpreted as normal body weight, and a BMI less than 18.5–underweight. Office blood pressure (BP) measurements were performed using a validated, semi-automated oscillometric device (OMRON HEM-CR24) with an appropriately sized cuff (22–32 cm). Prior to the procedure, study participants were instructed to avoid caffeine, tobacco, and exercise for 30 min before measurements. Following a 5-min rest period in a comfortable seated position, three sequential BP measurements were obtained 1–2 min apart, in accordance with the 2024 ESC guidelines. The final recorded BP was calculated as the average of the last two readings. Blood pressure (BP) categorization follows the Centers for Disease Control and Prevention (CDC) guidelines, defining high blood pressure as a systolic reading of 120 mmHg or greater, or a diastolic reading of 80 mmHg or greater (17). Moreover, hypertension was defined according to the guidelines set by the European Society of Cardiology and the European Society of Hypertension. According to these guidelines, normal blood pressure is considered to be below 120/70 mmHg. Elevated blood pressure falls within the range of 120–139 mmHg systolic and 70–89 mmHg diastolic. Hypertension is diagnosed when blood pressure is equal to or greater than 140/90 mmHg (11).

The quantitative variables were presented as median values with interquartile ranges (IQR) because they did not follow a normal distribution, as indicated by the Shapiro–Wilk test. Qualitative variables were described using their counts and corresponding percentages, the effect size of the percentage difference between compared periods was presented as Cramer’s *V* value and was calculated according to the formula *r* = $\frac{Z}{\sqrt{N}}$ , where Z denotes the standardized test statistic and N the total number of observations on which Z was based. To evaluate the differences in quantitative variables for the same students over the 2 years of observation, the Wilcoxon signed-rank test was used. Effect size *r* was presented as Cohen’s *r* value Additionally, to compare the percentage of medical students with high blood pressure across different BMI categories, we employed the chi-square test (McNemar’s test on paired nominal variables). The Kruskal–Wallis ANOVA test was conducted to evaluate the overall quality of life scores across different BMI categories. The analysis employed multivariate methods, particularly logistic regression, to examine the relation between a lower quality of life (defined as a total score below the median value of 75) and selected explanatory variables that were statistically significant in univariate analyses for both years. Internal consistency of the WHOQOL-BREF questionnaire was assessed using Cronbach’s alpha (α) coefficient. The internal consistency of the WHOQOL-BREF domains varied across study years, with α values ranging from 0.620 to 0.677 among first-year students and from 0.523 to 0.695 among second-year students. Compared with first-year students, second-year students showed lower α values. In the physical and psychological domains, indicating moderate overall internal consistency, with lower consistency observed in some domains. A significance level of *p* < 0.05 was established for all analyses. All statistical analyses were carried out using the Statistica 13.3 software package (TIBCO Software Inc., Palo Alto, CA, United States).

## Results

A total of 887 medical students were examined; however, in the second, subsequent year of the study, only 705 students (358 women and 347 men) participated in the project. Among the 887 students, 182 (20.5% of the initial group) either resigned from the university (*n* = 98) or did not consent to the current study (*n* = 84). Table 1 presents particular descriptive statistics for selected quantitative variables. As we can see, the value of Cohen’s *r* was nearly zero for each variable. Therefore, even if the Wilcoxon test results are statistically significant, the differences may be negligible. The median age of the students at the beginning of the study was 19.0 years, with an interquartile range (IQR) of 1.0 years.

**Table 1:** Descriptive statistics of the overall QoL scoring, BMI, and blood pressure measurements in the first and second university year of study.

Variable	The first year of observation			The second year of observation			*p*-value	Effect size (Cohen’s r)
	Number	Median	IQR	Number	Median	IQR		
BMI (kg/m^2^)	882	21.2	3.8	694	21.3	3.7	<0.001	0.03
SBP (mmHg)	872	114.0	16.0	531	118.0	15.0	0.002	0.01
DBP (mmHg)	872	72.0	12.0	531	73.0	13.0	0.003	0.04
Overall QoL	887	75.0	12.5	694	75.0	12.5	0.473	0.04

SBP, systolic blood pressure; DBP, diastolic blood pressure; BMI, body mass index; IQR, interquartile range; QoL, the overall quality of life scoring; p-value, statistical significance in the Wilcoxon test selected quantitative variables.

Detailed results of the study, including the number and percentage of students in selected categories of BMI value or blood pressure, are presented in Table 2. The frequency of obese or overweight students slightly increased in the second year of university study from the value of 12.9 to 13.6%. The same observation is related to the frequency of elevated blood pressure, a slight increase from the value of 67.4 to 72.9%. However, Cramer’s *V* was near zero for each variable, which confirmed a small effect size. Moreover, the highest percentage of high blood pressure was observed in obese students in both university years, and the observed difference was statistically significant, with a *p*-value < 0.0001 in the chi-square test for both years (Table 3.). The scoring of overall quality of life was the highest in students without obesity; however, the difference was not statistically significant (*p* = 0.645 and *p* = 0.192, respectively, in the first and second university year).

**Table 2:** The number and percentage of students in particular groups defined by BMI or blood pressure categories.

Variable	The first year		The second year		Results of chi^2^ test*	Effect size (Cramer’s *V*)
	*N*	(%)	*N*	(%)		
BMI categories						
Normal	772	87.1	603	86.4	<0.001	0.015
Overweight or obesity (BMI ≥ 25)	114	12.9	95	13.6		
Total group	886	100	698	100		
Blood pressure categories according to CDC criteria, 2024						
Normal (SBP < 120 and DBP < 80)	517	59.3	263	49.5	0.01	0.003
High blood pressure (SBP ≥ 120 or DBP ≥ 80)	355	40.7	268	50.5		
Total group	872	100	531	100		
Blood pressure categories according to ESC criteria, 2024						
Non-elevated (<120/70 mmHg)	277	31.8	139	26.2	<0.001	0.020
Elevated (120–139/70–89 mmHg)	588	67.4	387	72.9		
Hypertension (≥140/90 mmHg)	7	0.8	5	0.9		
Students with elevated BP or hypertension	595	68.2	392	73.8		
Total group	872	100	531	100		

SBP, systolic blood pressure; DBP, diastolic blood pressure; * McNemar’s test.

**Table 3:** The number and percentage of medical students with high blood pressure in particular BMI categories, and the results of the ANOVA Kruskal–Wallis test for the overall quality of life scoring obtained in both years of observation.

High blood pressure (CDC criterion) in the first year	BMI categories in the first year			
	Normal	Overweight	Obesity	Total
No	474 (62.9%)	36 (38.7%)	4 (20.0%)	514 (59.3%)
Yes	279 (37.1%)	57 (61.3%)	16 (80%)	352 (40.6%)
Total	753 (100%)	93(100%)	20 (100%)	866 (100%)
Result of Chi^2^ and p-value	33.3; *p* < 0.0001; Cramer’s *V* = 0.15			
High blood pressure (CDC criterion) in the second year	BMI categories in the second year			
No	251(54.8%)	7(11.9%)	3(37.5%)	261(49.7%)
Yes	207(45.2%)	52(88.1%)	5(62.5%)	264(50.3%)
Total	458 (100%)	59(100%)	8(100%)	525(100%)
Result of Chi^2^ and *p*-value	39.03; *p* < 0.0001; Cramer’s *V* = 0.27			
Quality of Life	Mean value ± SD / Me(IQR)			
Overall scoring in the first year	68.2 ± 17.9/75(13)	63.4 ± 20.4/62.5(25)	63.1 ± 18.8/62.5 (31)	67.6 ± 18.2/75(12.5)
Results of the ANOVA Kruskal–Wallis test	0.87; *p* = 0.645			
Overall scoring in the second year	68.8 ± 17.5/75(12.5)	68.9 ± 20.3/62.5(25)	61.4 ± 20.9/62.5(31)	68.7 ± 17.9/75(12.5)
Results of the ANOVA Kruskal–Wallis test	3.29; *p* = 0.192			

BMI, body mass index; p, statistical significance in chi-square test; SD, standard deviation.

Table 4 presents the percentage of obese or overweight students in the first and second university years, categorized by selected groups defined by explanatory variables such as sex, socioeconomic status, and lifestyle. The frequency of obese or overweight medical students was significantly higher in male than female students, in students with the poorest financial status, in those who preferred meals containing animal protein in daily diet, and in students with higher blood pressure. Those observations revealed both university years. Moreover, the percentage of obese or overweight students was higher in those who declared lower consumption of vegetables or fruits and wholemeal bread, groats, and dry leguminous vegetables in their daily diet, in hazardous users of alcohol, and in students with lower physical activity. Additionally, obesity or being overweight is more often associated with students who have a recognized chronic disease and have lower physical activity. However, no significant effect size (value of Cramer’s *V*) was observed for the variables compared.

**Table 4:** The percentage of obese or overweight students in a particular group defined by explanatory variables in both university years.

Explanatory variable		The first year		Effect size	The second year		Effect size
		% of overweight or obesity	*p*		% of overweight or obesity	*p*	
Sex	Male	21.4	<0.001	0.17	25.7	<0.001	0.03
	Female	8.6			8.0		
Alcohol consumption	Low risk	11.0	0.052	0.04	11.9	0.386	0.09
	Hazard use	15.8			14.4		
Current financial situation	Average or poor	16.9	0.029	0.07	19.0	0.009	0.07
	Good or very good	11.4			11.2		
Current place of residence during studies at university	Family home	12.1	0.695	0.01	16.7	0.139	0.01
	Dormitory/rented flat or room	13.1			12.1		
Current cigarette smoking (traditional or electronic)	Never or in the past, not now	17.2	0.132	0.05	13.2	0.894	0.01
	Yes, still	12.2			13.9		
Frequency of physical activity	Yes, regularly or less often	10.7	0.222	0.04	11.2	0.067	0.01
	Not at all	13.7			16.1		
Current health status	Average or poor	17.9	<0.001	0.12	15.2	0.196	0.06
	Good or very good	9.1			11.8		
Ever diagnosed with a chronic disease	Yes	18.3	0.004	0.09	17.0	0.138	0.05
	No	10.9			12.4		
High blood pressure (CDC criterion)	No	7.8	<0.001	0.18	3.8	<0.001	0.19
	Yes	20.7			21.6		
Number of meals per day	At least 3 a day	12.6	0.553	0.01	11.3	0.569	0.01
	Less than 3	14.7			13.4		
Number of meals containing animal protein	In all meals, at least 75% of meals	15.4	0.004	0.09	16.5	<0.001	0.08
	Fewer meals	8.8			7.3		
Frequency of milk or cheese consumption	Daily at least once a day	13.4	0.560	0.19	18.7	0.053	0.15
	Less often	12.0			12.0		
Frequency of vegetable or fruit consumption	Daily at least twice a day	10.4	0.001	0.10	11.5	0.068	0.05
	Less often	18.2			16.6		
Frequency of raw vegetable or fruit consumption	Daily, at least 75% of meals	10.3	0.041	0.06	9.8	0.151	0.02
	Less often	14.9			14.1		
Frequency of wholemeal bread, groats, and dry leguminous vegetables consumption	Daily, at least in 1 meal, at least 75% of meals	11.9	0.241	0.03	11.6	0.154	0.03
	Less often	14.6			15.3		

p, statistical significance in chi square test and Cramer’s *V* as the effect size.

Table 5 presents the results of logistic regression for the relationship between declared, overall quality of life, and selected explanatory variables in total subjects and in separate BMI categories (normal weight and obese or overweight students, respectively). It has been documented that statistically significant importance to the worst quality of life was attributed to the poorest financial situation (OR = 1.669 and OR = 1.910, in the first- and second-year, respectively) and ever-diagnosed chronic disease (OR = 2.228 and OR = 2.153, in the first- and second-year, respectively). Stratification by BMI categories revealed similar results to those obtained in the total population. In students with a normal weight, a worse financial situation, and the occurrence of chronic disease were independent predictors of the worst QoL. In the obese or overweight students, no statistical significance was documented.

**Table 5:** Results of logistic regression.

Explanatory variable	First-year students			Second-year students		
	OR	95%CI	*p*	OR	95%CI	*p*
The worst quality of life (QoL scoring < 75) in the total group of medical students						
Sex (1 = Female)	1.282	0.934–1.760	0.125	0.910	0.630–1.313	0.614
High blood pressure – CDC (1 = yes)	0.984	0.734–1.320	0.914	0.949	0.659–1.365	0.776
Poorest financial situation (1 = yes)	1.669	1.224–2.276	0.001	1.910	1.247–2.926	0.003
Ever diagnosed with a chronic disease (1 = yes)	2.228	1.616–3.072	<0.001	2.153	1.457–3.184	<0.001
Number of meals containing animal protein (1 = In all meals, at least 75% of meals)	1.278	0.953–1.716	0.102	0.935	0.645–1.354	0.720
Frequency of vegetable or fruit consumption (1 = Daily at least twice a day)	1.336	0.990–1.805	0.059	0.641	0.435–0.944	0.024
The worst quality of life (QoL scoring < 75) in normal weight students (BMI < 25)						
Sex (1 = Female)	1.430	1.004–2.035	0.047	0.963	0.648–1.430	0.851
High blood pressure (1 = yes)	0.996	0.723–1.373	0.981	1.022	0.691–1.511	0.915
Poorest financial situation (1 = yes)	1.727	1.232–2.422	0.002	1.974	1.234–3.159	0.005
Ever diagnosed with a chronic disease (1 = yes)	2.250	1.582–3.200	<0.001	2.045	1.342–3.117	0.001
Number of meals containing animal protein (1 = In all meals, at least 75% of meals)	1.260	0.920–1.725	0.150	0.917	0.618–1.360	0.667
Frequency of vegetable or fruit consumption (1 = Daily at least twice a day)	1.283	0.921–1.787	0.140	0.725	0.474–1.107	0.137
The worst quality of life (QoL scoring < 75) in obese or overweight students (BMI ≥ 25)						
Sex (1 = Female)	1.158	0.504–2.659	0.730	0.452	0.138–1.474	0.188
High blood pressure – CDC (1 = yes)	1.633	0.686–3.888	0.268	1.479	0.281–7.782	0.644
Poorest financial situation (1 = yes)	1.088	0.479–2.470	0.841	1.773	0.542–5.799	0.343
Ever diagnosed with a chronic disease (1 = yes)	1.500	0.645–3.485	0.346	2.833	0.822–9.765	0.099
Number of meals containing animal protein (1 = In all meals, at least 75% of meals)	1.507	0.579–3.923	0.401	0.346	0.073–1.635	0.180
Frequency of vegetable or fruit consumption (1 = Daily at least twice a day)	1.239	0.557–2.753	0.599	0.368	0.116–1.167	0.090

QoL, quality of life; OR, odds ratio; 95% CI, 95% confidence interval; p, statistical significance.

## Discussion

During a two-year follow-up of first- and second-year medical students (*n* = 882 students and *n* = 694, respectively), we observed a slight statistically significant increase in the mean value of BMI (*p* < 0.001). The prevalence of overweight or obesity (BMI ≥ 25 kg/m^2^) was 12.9% in the first year and 13.6% in the second year. Excess body weight was significantly more common among male than female medical students. These findings are consistent with a systematic review and meta-analysis including 47.455 medical students from diverse geographical regions, which reported a combined prevalence of overweight and obesity of 24%. The meta-regression analysis indicated a temporal increase in excessive body weight and a higher prevalence among male students, confirming the trends observed in our cohort group (18).

Our study aimed to assess the relationship between overweight or obesity and quality of life (QoL) among medical students in Poland, and to identify its determinants over a longitudinal period. Overweight and obesity remain major global public health concerns. According to a report published in The Lancet Diabetes & Endocrinology, approximately 2.11 billion adults aged ≥25 and 493 million individuals aged 5–24 are affected by excess body weight.

Longitudinal evidence on the determinants of overweight and obesity and their impact on QoL in young populations remains limited. In light of the updated 2025 obesity definition proposed by the Lancet Commission (19), as well as the 2026 dietary guidelines issued by the US Department of Health and Human Services and the US Department of Agriculture (20), continuation of these relationship assessments is warranted.

Additionally, we observed a 5.5% increase in the elevated blood pressure between the first and second year of follow-up. The highest rates of elevated blood pressure were consistently found among those with obesity. However, elevated blood pressure was not a statistically significant predictor of reduced QoL in our cohort. Other studies have reported similar findings regarding blood pressure abnormalities among medical students. Research conducted by Ali N. et al. among students in Bangladesh indicated that the frequency of general and abdominal obesity was 8.3 and 20.7%, respectively. Moreover, 26% of individuals were overweight. No statistically significant sex differences were observed; however, the prevalence of general obesity was slightly higher among females. Concerning blood pressure, the prevalence of prehypertension and hypertension was 37.8 and 10.2%, respectively. The authors also noted that both prehypertension and hypertension rates were significantly higher in the obese compared with the normal-weight and overweight groups (21). Similar findings were reported in a study conducted among medical students in India (22). In that study, a positive correlation with BMI and systolic and diastolic blood pressure was identified. The prevalence of overweight or obesity according to BMI categories was 12.6 and 46.5%, respectively. Furthermore, a study conducted among a large group of university students in West Africa (*N* = 2030) indicated that the overall prevalence of hypertension was 6.0%, with significantly higher rates among male students (6.8%) than female students (2.7%). Overall, 7.3% of students were classified as overweight or obese based on BMI criteria. Being overweight or obese was identified as a significant predictor of elevated blood pressure (OR = 3.75; 95% CI = 2.13–6.59) (23).

The presence of chronic diseases (illnesses lasting 6 months or longer) among medical students is a significant concern as society often expects physicians to exemplify a healthy lifestyle. Our study found a link between a history of chronic disease and a lower quality of life among medical students. For instance, Prazeres et al. (24) reported that 23% of 309 medical students in Portugal were affected by chronic diseases, with 40% having a positive family history of such conditions. Research shows that students with chronic diseases experience a significantly lower QoL and higher levels of anxiety compared to their peers without health issues. In a study by Couinho et al. (25), students with chronic diseases (*N* = 232) reported a lower QoL than those without diseases (*N* = 115). This finding aligns with observations from our previous study in the POLLEK series (26), where we noted that medical students with chronic diseases had significantly lower scores in the general, somatic, and psychological aspects of QoL.

Psychosocial pathways may also link excess weight with perceived well-being; recent evidence among children with overweight or obesity indicates that weight misperception, adverse social experiences and bullying-particularly among boys-may accompany excess body weight, although differences in age and setting limit direct comparison with university students (27).

Sociodemographic characteristics are important determinants of both obesity and QoL among university students. Factors such as sex, financial situation, place of residence, and living conditions may influence health behaviors, access to resources, and perceived well-being. In our study, male students were more likely to be overweight or obese, which is in line with a recent multicenter analysis among university students demonstrating a higher prevalence of excess body weight in men and sex-related differences in health behaviors (8, 28). Lower socioeconomic status has been linked to poorer dietary quality, limited access to physical activity opportunities, and higher stress levels, all of which may contribute to increased obesity risk and reduced QoL (29). The interplay between sociodemographic factors and obesity suggests a complex, multidimensional pathway through which these variables jointly influence QoL outcomes. Therefore, understanding these relationships is essential for identifying vulnerable subgroups and designing targeted interventions aimed at improving both health status and quality of life among medical students.

The financial status of young people who are beginning to live independently can vary based on the level of financial support from relatives and their money management skills. Our study showed a significant negative impact of poor financial status on the QoL of medical students in both the first- and second- year of observation. Riaz et al. (30) demonstrated that socioeconomic status is one of the strongest determinants of dietary quality among university students, finding that students from higher-income households could afford to consume more fruits and vegetables, lean proteins, and generally high-quality foods. Also, financial factors indicated a strong association with the occurrence of depression, anxiety, and burnout symptoms among Lebanese students (31). Both low or moderate socioeconomic status and insufficient or moderate pocket money for medical students at Chosun University predict lower QoL in the environmental domain (32). Research confirms that socioeconomic factors, and above all, a poor financial situation, can negatively impact students’ quality of life.

Lifestyle, particularly diet, physical activity, tobacco use, and alcohol consumption, play a crucial role in both the development of overweight and obesity and QoL outcomes. Numerous studies indicate that unhealthy lifestyle patterns increase the risk of excessive body weight and are associated with poorer QoL among young adults. For instance, Taher et al. (33) demonstrated a significant association between dietary habits and BMI. Analysis conducted among 320 medical students revealed that 15% of overweight and 3.1% of obese individuals reported frequent consumption of red meat, fried and processed foods, and sweets, with lower intake of fruits and vegetables. Furthermore, a study among 200 medical students in India found that 44.5% were overweight or obese, and there was a significant correlation between lower frequency of physical activity and obesity (*p* = 0.002) (34). Also, Ilić et al. (35) found, in a study, among 2,452 medical students, that smoking status was a significant positive predictor of overweight and obesity. The odds of being overweight or obesity was 1.371 times higher for smokers than non-smokers (95% CI: 1.060–1.774; *p* = 0.016). Alcohol abuse and smoking are often ways of coping with stress resulting from students’ unsatisfactory QoL. Such behaviors are considered risky health behaviors that increase the risk of overweight or obesity and non-communicable diseases. Tran et al. (36) indicated that overweight and risky alcohol use (33.78% in 148 college students) was among the top co-occurrences of cardiovascular risk factors. Authors indicate that medical students may have difficulty translating theoretical nutritional knowledge into practical eating habits, which could negatively affect their health, predispose them to overweight and obesity, and, as a result, reduce the quality of life (37). During a two-year observation, we noticed a higher frequency of excess body mass in students who preferred meals containing animal protein and had lower consumption of bread, groats, and dry leguminous in their daily diet. According to the meta-analysis of 95 studies (38), a poor diet in fruits and vegetables is associated with increased risk of developing overweight or obesity and with weight gain, cardiovascular disease, and cancer. Even 5.6 and 7.8 million premature deaths worldwide in 2013 could be connected with low fruit and vegetable intakes (below 500 and 800 g/day, respectively). In our study, 18.2% of the 887 and 16.6% of the 705 study population who reported consuming vegetables or fruits less than once a day were overweight or obese according to BMI. Taking into account, mentioned in the World Health Organization Health Inequality Data Repository (39), the dimensions of inequality analysis in adult health and nutrition, alcohol consumption, and tobacco smoking, such as age, economic status, sex, and education, with our study, we wanted to provide our own data that would show the convergence of these phenomena. Our goal was also to draw attention to the possibility of controlling those factors that significantly affect the quality of life, assessing both the latest situation of medical students and changes over time.

Building on this evidence, it is essential to further examine the combined impact of dietary patterns and physical activity levels on both the risk of overweight/obesity and the quality of life of medical students over time. Such an approach may help to better understand modifiable determinants of health and inform targeted preventive strategies in this population.

## Strengths and limitations of this study

The main strength of this prospective study is that we observed a cohort of medical students during their first and second years of education, following determinants such as obesity or overweight that affect QoL. It is also worth noting that in both the first- and second- year, we observed a high participation rate (approximately 84% response rate at T1 and T2). Although the number of participants decreased from 887 at T1 to 705 at T2, corresponding to approximately 20.5% attrition between the first and second assessments, the study nevertheless allowed us to monitor changes over time. Importantly, the follow-up response rate remained high (79.5%). However, the loss to follow-up may have introduced attrition bias, which cannot be completely excluded and should therefore be considered when interpreting the longitudinal findings.

Referring to the previously published results of the POLLEK study (40), the study group was more than doubled, which undoubtedly had an impact on strengthening the reliability and credibility of the obtained results. The study highlights the impact of weight disorders on the medical students’ quality of life. To screen students for their nutritional status, we used the BMI. This anthropometric indicator has some limitations, including the inability to distinguish between fat and muscle tissue. However, it is widely used in scientific research.

Moreover, the overall WHOQOL-BREF score was dichotomised using the sample median to distinguish between relatively lower and higher quality of life. While this approach facilitated the interpretation of results and enabled the use of logistic regression models, it may have resulted in some loss of information and reduced statistical power compared with analyses treating QoL as a continuous variable. Therefore, the observed associations should be interpreted as factors related to relatively lower quality of life rather than changes across the full spectrum of QoL scores.

The data obtained provide an opportunity for early monitoring of young people’s health and may be important for formulating practical recommendations that can be used to implement early prevention in this group. We are aware that this is a single-center study, which has a limited generalizability of the study results to medical students in the context of quality of life determinants. Finally, this is not a full follow-up observation; however, the next stage of the study is planned in the same group of students in their 4th and 5th year of medical studies.

## Conclusion

The results of our study confirmed the slight increase in the prevalence of overweight and obesity among medical students during the 2 years of observation. We identified the following modifiable and non-modifiable associated factors towards overweight or obesity among medical students: male gender, low financial status, and elevated blood pressure. The aforementioned sociodemographic and lifestyle determinants can significantly impact their QoL. Our observations suggest that early prevention of overweight and obesity can be useful to improve quality of life.

## Data Availability

The raw data supporting the conclusions of this article will be made available by the authors, without undue reservation.
